# AIB-OR: Improving Onion Routing Circuit Construction Using Anonymous Identity-Based Cryptosystems

**DOI:** 10.1371/journal.pone.0121226

**Published:** 2015-03-27

**Authors:** Changji Wang, Dongyuan Shi, Xilei Xu

**Affiliations:** 1 National Pilot School of Software, Yunnan University, Kunming, China; 2 Yunnan Key Laboratory of Software Engineering, Yunnan University, Kunming, China; 3 School of Information Science and Technology, Sun Yat-sen University, Guangzhou, China; 4 Guangdong Key Laboratory of Information Security Technology, Sun Yat-sen University, Guangzhou 510275, China; Nanyang Technological University, SINGAPORE

## Abstract

The rapid growth of Internet applications has made communication anonymity an increasingly important or even indispensable security requirement. Onion routing has been employed as an infrastructure for anonymous communication over a public network, which provides anonymous connections that are strongly resistant to both eavesdropping and traffic analysis. However, existing onion routing protocols usually exhibit poor performance due to repeated encryption operations. In this paper, we first present an improved anonymous multi-receiver identity-based encryption (AMRIBE) scheme, and an improved identity-based one-way anonymous key agreement (IBOWAKE) protocol. We then propose an efficient onion routing protocol named AIB-OR that provides provable security and strong anonymity. Our main approach is to use our improved AMRIBE scheme and improved IBOWAKE protocol in onion routing circuit construction. Compared with other onion routing protocols, AIB-OR provides high efficiency, scalability, strong anonymity and fault tolerance. Performance measurements from a prototype implementation show that our proposed AIB-OR can achieve high bandwidths and low latencies when deployed over the Internet.

## Introduction

The rapid development of network technology has made anonymous communication an increasingly important security requirement for many network applications [1]. While end-to-end encryption can protect the data content of communications from unauthorized access, it does not conceal all the relevant information that two communicating parties are communicating. For example, routing information is still transmitted in the clear because routers need to know packets’ destinations in order to route them in the right direction. Traffic analysis can also be done by watching particular data moving through a network, by matching the amount of data, or by examining coincidences, such as connections opening and closing at about the same time.

In many situations, it is highly desirable or indispensable for users to be able to preserve the communications anonymity. For example, an abrupt change in the traffic pattern may indicate some forthcoming activities in a tactical military communication network. This can be extremely dangerous in that adversaries can easily identify critical network nodes and then launch targeted attacks on them. In addition, people have a strong desire to remain anonymous when pursuing sensitive information in order to avoid unnecessary trouble.

Over the years, a large number of anonymity networks have been proposed and some have been implemented. Among them, onion routing has been widely employed as an infrastructure for private communication over a public network.

### Related Work

#### Identity-Based Cryptography

To simplify certificate management in tradition public key infrastructure, Shamir [2] first introduced the concept of identity-based public key cryptography, where an entity’s public key can be publicly computed from his recognizable identity information, such as a complete name or an e-mail address, while the corresponding private key is generated by a trusted third party named as private key generator (PKG).

The first practical and secure identity-based encryption (IBE) scheme was constructed from bilinear pairings by Boneh and Franklin [3]. Since then, various IBE schemes, identity-based signature schemes and identity-based key agreement (IBKA) protocols have been proposed [4].

For example, considering a situation where a sender would like to encrypt a message for *t* receivers, the sender must encrypt the message *t* time using conventional IBE schemes. To improve the performance, Baek et al. [5] introduced the notion of multi-receiver IBE scheme, and proposed an efficient provably secure multi-receiver IBE scheme from bilinear pairings. To guarantee receiver’s privacy, Boyen and Waters [6] proposed an anonymous IBE scheme, where the ciphertext does not leak the identity of the recipient.

Later, Fan et al. [7] introduced the concept of anonymous multi-receiver IBE (AMRIBE) scheme and proposed an efficient AMRIBE scheme from bilinear parings. In an AMRIBE scheme, one can examine whether himself is a selected receiver or not. Nobody, except the sender, knows who the other selected receivers are. Subsequently, Chien [8] pointed out that Fan et al.’s AMRIBE scheme only provides receiver anonymity for outsider attackers or non-selected receivers, and presented an improved AMRIBE scheme. However, only heuristic arguments for security proofs are presented. Tseng et al. [9] proposed a new AMRIBE scheme that was proved to be semantically secure against adaptive chosen ciphertext attacks in the random oracle model under the Gap-BDH assumption.

Sakai et al. proposed the first non-interactive IBKA protocol from bilinear pairing, where the established key consists of only one participant’s identity-based private key and the other participant’s identity. Thus, the established key can not be used as a session key because it always establishes the same (secret) key for the same entities in each run of the protocol. Kate et al. [10] extended Sakai et al.’s IBKA protocol, and proposed an identity-based one-way anonymous key agreement (IBOWAKE) protocol to provide unconditional anonymity for participants by replacing the identities of the participants by pseudonyms. Unfortunately, Kate et al. IBOWAKE protocol is insecure against man-in-the-middle (MIMA) attack because it can not authenticate the communication entities.

#### Certificateless Cryptography

The concept of certificateless public key cryptography was first introduced by Al-Riyami and Paterson [11], which combines the advantages of traditional certificate-based public key cryptography and identity-based public key cryptography. In a certificateless cryptosystem, the key generation center (KGC) does not have access to the entity’s private key, the KGC derives a partial private key from the entity’s identity and the master secret key. The entity then combines the partial private key with some secret information to generate the actual private key. Thus, certificates are not considered necessary anymore to guarantee the authenticity of public keys in traditional certificate-based public key cryptography, and at the same time the private key is not fully determined by the KGC to prevent the inherent key escrow problem in identity-based public key cryptography.

Certificateless public key cryptography has received a significant attention in recent years, several certificateless encryption schemes, certificateless signature schemes and certificateless key agreement protocols were presented. For example, Catalano et al. [12] introduced the concept of anonymous certificateless key agreement and proposed two constructions.

#### Attribute-Based Encryption

Attribute-based encryption (ABE) was first introduced by Sahai and Waters [13] with the original goal of providing an error-tolerant IBE that uses biometric identities. ABE can be viewed as an extension of the notion of IBE in which user identity is generalized to a set of descriptive attributes instead of a single string specifying the user identity. Compared with IBE, ABE has significant advantage as it achieves flexible one-to-many encryption instead of one-to-one, it is envisioned as a promising tool for addressing the problem of secure and fine-grained data sharing and decentralized access control.

ABE have drawn extensive attention from both academia and industry in recent years, many ABE schemes have been proposed [14–16] and several cloud-based secure systems using ABE have been developed [17–19]. There are two types of ABE depending on which of private keys or ciphertexts that access policies are associated with.

In a key-policy attribute-based encryption (KP-ABE) system, ciphertexts are labeled by the sender with a set of descriptive attributes, and users’ private keys are issued by the trusted attribute authority are associated with access policies (also called access structures) that specify which type of ciphertexts the key can decrypt. In a ciphertext-policy attribute-based encryption (CP-ABE) system, when a sender encrypts a message, they specify a specific access policy in terms of access structure over attributes in the ciphertext, stating what kind of receivers will be able to decrypt the ciphertext. Users possess sets of attributes and obtain corresponding secret attribute keys from the attribute authority, such a user can decrypt a ciphertext if his/her attributes satisfy the access policy associated with the ciphertext.

#### Onion Routing

Onion routing was first proposed by Reed, Syverson and Goldschlag [20, 21]. In onion routing, for a given connection, the sender selects a sequence of routers, known as a circuit, that will be used to forward the sender’s traffic. The sender establishes a circuit by first directly opening a circuit with the first router, and then iteratively extending the circuit by sending message over the existing circuit. Messages are encrypted with the key of each router in the circuit in the reverse order that the routers appear. Like someone peeling an onion, each onion router removes a layer of encryption to uncover routing instructions, and sends the message to the next router where this is repeated. This prevents these intermediary nodes from knowing the origin, destination, and contents of the message.

In the original onion routing protocol [22], each onion router is equipped with a pair of public and private key. The source uses the public keys of the intermediate routers with the top layer encrypted with the public key of the router immediately next to the source. The intermediate routers then use their own corresponding private keys to decrypt the packet and obtain the information about the next hop in the network. The packets thus routed and forwarded by each intermediate genuine node, eventually reach the destination. The advantage here is that if any one of the routers is compromised by an adversary, even then, the other components remain beyond the reach, because of being encrypted using a different public key. However, it is evident that a sender is required to encrypt a message as many times as is the number of intermediate onion routers.

Kate et al. [10] presented a pairing-based onion routing (PB-OR) protocol by using an IBOWAKE protocol. Catalano et al. [12] then proposed a certificateless onion routing (CL-OR) protocol by using an anonymous certificateless key agreement protocol. Later, Catalano et al. [23] proposed a fully non-interactive onion routing protocol with forward secrecy by using a forward-secure IBE scheme. Recently, Doshi et al. [24] proposed an onion routing circuit construction called attribute-based onion routing (AB-OR) using the Bethencourt et al. CP-ABE scheme [15]. Here the access policy is boolean formula over routers’ identities and the access policy is sent in the clear along with the ciphertext. Thus, AB-OR provides neither recipient anonymity nor route anonymity.

### Motivation and Our Contributions

Compared to the original onion routing protocol, onion routing protocols proposed in recent years have greatly improved in terms of efficiency and anonymity. However, there are still three drawbacks in the existing onion routing protocols.

- Failure tolerance is relatively poor. The palsy of any one of the intermediate onion router will result in the palsy of the entire onion routing process.
- Recipient anonymity is not strong enough. The last onion router will know the identity of the recipient.
- Communication anonymity is weak. Each intermediate onion router needs to know the identity of the next hop router on the path, which impairs the communication anonymity.

In this paper, we first improve Kate et al.’s IBOWAKE protocol [10] and Tseng et al.’s AMRIBE scheme [9], then we propose a new onion routing circuit construction called anonymous identity-based onion routing (AIB-OR) by integrating our improved IBOWAKE protocol with AMRIBE scheme in onion routing circuits construction. Compared to PB-OR and CL-OR, our proposed AIB-OR achieves both efficiency improvement and anonymity enhancement. This paper makes three primary contributions in the field of anonymous communication.

- The efficiency of onion routing circuit construction is improved. The performance of AIB-OR surpasses those of PB-OR and CL-OR, requiring significantly less computation and fewer network communications. Unlike existing onion routing protocols, Our proposed AIB-OR only requires the sender to encrypt the message twice, irrespective of the number of intermediate onion routers.
- Failure tolerance of onion routing circuit construction is provided. The sender can select any one of the path through which to forward the packet out of the multiple paths at its disposal in the proposed AIB-OR.
- Anonymity of onion routing circuit construction is enhanced. The sender, the recipient and the intermediate onion routers are anonymous to others, no one knows the real identities and location of the sender, the intermediate onion routers, or the recipient. Adversaries cannot trace a packet flow back to its sender or the recipient. Nobody, except the sender, knows the real routing path between the sender and the recipient.

### Paper Organization

The rest of this paper is organized as follows. Some necessary preliminary work and our improved IBOWAKE protocol and AMRIBE scheme are introduced in Section 2. The proposed AIB-OR model and construction are described in Section 3. Efficiency and security analysis of our AIB-OR are discussed in Section 4. Performance test of PB-OR and our AIB-OR are explained in Section 5. Finally, we conclude our work in Section 6.

## Preliminary Work

### Notations

To facilitate further description, we introduce notations in Table 1.

**Table 1:** Notations.

Symbol	Description
$x\overset{\text{\$}}{\leftarrow }\text{S}$	Pick an element *x* uniformly at random from the set **S**
*κ*	The system security parameter
Π	A semantically secure symmetric encryption scheme
len	The key length of Π
E_*k*_(*m*)	Encrypt a message *m* under Π with a session key *k* ∈ {0, 1}^len^
D_*k*_(*c*)	Decrypt a ciphertext *c* under Π with a session key *k* ∈ {0, 1}^len^
*H*_0_	Hash function $H_{0}:{\left\{0,1\right\}}^{*}\to G_{1}^{*}$
*H*_1_	Hash function $H_{1}:{\text{G}}_{2}\to {\text{Z}}_{q}^{*}$
*H*_2_	Hash function $H_{2}:Z_{q}^{*}\to {\left\{0,1\right\}}^{len}$
*H*_3_	Hash function *H*_3_:{0, 1}^*^ → {0, 1}^2len^

### Bilinear Group Generator and Complexity Assumptions

**Definition 1** *The bilinear group generator 𝒢 is an algorithm that takes as input a security parameter κ and outputs a bilinear group* (*q*, **G**_1_, **G**_2_, *ê*, *P*), *where q is a prime of size* 2^*κ*^, **G**_1_ and **G**_2_ *are cyclic groups of order q*, *P is a generator of* **G**_1_, *and ê*:**G**_1_×**G**_1_ → **G**_2_ *is a bilinear map with the following properties:*

- *Bilinearity: For* $a,b\overset{\$}{\leftarrow }{\text{Z}}_{q}^{*}$, *we have ê*(*aP*, *bP*) = *e*(*P*, *P*)^*ab*^.
- *Non-degeneracy:* *ê*(*P*, *P*) *is a generator of* **G**_2_.
- *Computability: For* $P_{1},P_{2}\overset{\$}{\leftarrow }{\text{G}}_{1}$, *there is an efficient algorithm to compute ê*(*P*_1_, *P*_2_).

**Definition 2 (Bilinear Diffie-Hellman Assumption)** *The BDH assumption in a prime order bilinear group* (*q*, **G**_1_, **G**_2_, *ê*, *P*) *generated by* 𝒢(1^*κ*^) *is that if a tuple* $\langle P,aP,bP,cP\rangle \in {\text{G}}_{1}^{\left(4\right)}$ *is given for unknown* $a,b,c\overset{\$}{\leftarrow }{\text{Z}}_{q}^{*}$, *there is no probabilistic polynomial-time (PPT) adversary 𝒜 can compute* *ê*(*P*, *P*)^*abc*^ ∈ **G**_2_ *with non-negligible advantage* [3].

**Definition 3 (Decisional Bilinear Diffie-Hellman Assumption)** *The DBDH assumption in a prime order bilinear group* (*q*, **G**_1_, **G**_2_, *ê*, *P*) *generated by* 𝒢(1^*κ*^) *is that if a tuple* $\langle P,aP,bP,cP,T\rangle \in {\text{G}}_{1}^{\left(4\right)}\times {\text{G}}_{2}$ *is given for unknown* $a,b,c\overset{\$}{\leftarrow }{\text{Z}}_{q}^{*}$ *and* $T\overset{\$}{\leftarrow }{\text{G}}_{2}$, *there is no PPT adversary 𝒜 can decide whether T* = *ê*(*P*, *P*)^*abc*^ *with non-negligible advantage* [4].

**Definition 4 (Gap Bilinear Diffie-Hellman Assumption)** *The Gap-BDH assumption in a prime order bilinear group* (*q*, **G**_1_, **G**_2_, *ê*, *P*) *generated by* 𝒢(1^*κ*^) *is that if a tuple* $\langle P,aP,bP,cP\rangle \in {\text{G}}_{1}^{\left(4\right)}$ *is given for unknown* $a,b,c\overset{\$}{\leftarrow }{\text{Z}}_{q}^{*}$, *there is no PPT adversary 𝒜 can compute ê*(*P*, *P*)^*abc*^ ∈ **G**_2_ *with the help of the DBDH oracle with non-negligible advantage. The DBDH oracle means that given a tuple* $\langle P,aP,bP,cP,T\rangle \in {\text{G}}_{1}^{\left(4\right)}\times {\text{G}}_{2}$, *outputs 1 if* *T* = *ê*(*P*, *P*)^*abc*^ *and 0 otherwise* [9].

### Our Improved IBOWAKE Protocol

Kate et al. [10] proposed an IBOWAKE protocol that was proved to be secure in the random oracle model under the BDH assumption. The core idea of Kate et al.’s IBOWAKE protocol is to replace the identity hashes with pseudonyms generated by users, and each user can randomly generate many possible pseudonyms and the corresponding private keys.

To avoid impersonation and MIMA attacks, it is desirable that only the pseudonym with valid certificate can be used as an encryption key during an anonymous communication session. For privacy purposes, we require that the PKG will not know the real pseudonym of an entity and the corresponding certificate. Our improved IBOWAKE protocol is described as follows.

- **Setup**: The PKG runs 𝒢(1^*κ*^) to get (*q*, **G**_1_, **G**_2_, *ê*, *P*), chooses $s\overset{\$}{\leftarrow }{\text{Z}}_{q}^{*}$, computes *P*_*pub*_ = *sP* ∈ **G**_1_. Finally, the PKG sets the master secret key *msk* = *s* and the system parameters *mpk* = ⟨*q*, **G**_1_, **G**_2_, *ê*, *P*, *P*_*pub*_, *H*_0_⟩.
- **Extract**: Given a user’s identity ID, the PKG computes *Q*_ID_ = *H*_0_(ID) and *d*_ID_ = *sQ*_ID_. Finally, the PKG sends the user’s private key *d*_ID_ to the user via a secure channel.
- **PNGen**: An entity X chooses $k_{X}\overset{\$}{\leftarrow }{\text{Z}}_{q}^{*}$, computes PN_*X*_ = *k*_*X*_ *P* and *sk*_PN_*X*__ = *k*_*X*_ *P*_*pub*_ = *s*PN_*X*_. Finally, the entity X sets PN_*X*_ and *sk*_PN_*X*__ as his pseudonym and the corresponding private key, respectively.
- **BlindCert** An entity X with a pseudonym PN_*X*_ chooses $r_{X}\overset{\$}{\leftarrow }{\text{Z}}_{q}^{*}$, generates a masked pseudonym by computing ${\text{PN}}_{X}^{\prime }=r_{X}H_{0}({\text{PN}}_{X})$, X then sends ${\text{PN}}_{X}^{\prime }$ to the PKG. The PKG computes $\sigma_{X}^{\prime }=s{\text{PN}}_{X}^{\prime }$ and sends the signature $\sigma_{X}^{\prime }$ to X. Upon receiving $\sigma_{X}^{\prime }$, X verifies $\sigma_{X}^{\prime }$ by checking $\hat{e}(\sigma_{X}^{\prime },P)=\hat{e}({\text{PN}}_{X}^{\prime },P_{pub})$. If the equation holds, X then computes $\sigma_{X}=r_{X}^{-1}\sigma_{X}^{\prime }=sH_{0}({\text{PN}}_{X})$, and obtains his pseudonym certificate 〈PN_*X*_, *σ*_*X*_〉. Anyone can verify entity X’s pseudonym certificate 〈PN_*X*_, *σ*_*X*_〉 by testing *ê*(*σ*_*X*_, *P*) = *ê*(*H*_0_(PN_*X*_), *P*_*pub*_).
- **Key Agreement**: Suppose Alice wants to perform a session key agreement with Bob. Alice knows Bob’s identity ID_*B*_ and wishes to remain anonymous to Bob, Alice and Bob perform the following steps:
  - Alice generates her pseudonym PN_*X*_ and gets her pseudonym certificate 〈PN_*A*_, *σ*_*A*_〉 by performing the **PNGen** algorithm and the **BlindCert** algorithm, respectively.
  - Alice computes the session key *K*_*A*, *B*_ = *ê*(*sk*_PN_*A*__, *Q*_ID_*B*__). Alice then sends her pseudonym certificate 〈PN_*A*_, *σ*_*A*_〉 to Bob.
  - Upon receiving Alice’s pseudonym certificate 〈PN_*A*_, *σ*_*A*_〉, Bob verifies Alice’s pseudonym certificate by testing *ê*(*σ*_*A*_, *P*) = *ê*(*H*_0_(PN_*A*_), *P*_*pub*_). If the equation holds, Bob then computes the corresponding session key *K*_*A*, *B*_ = *ê*(PN_*A*_, *d*_ID_*B*__) by using his private key *d*_ID_*B*__.

Note that the **BlindCert** algorithm is in fact a blind Boneh-Lynn-Shacham (BLS) signature scheme [25], which is proved to be existentially unforgeable under adaptive chosen-message attacks under the computational Diffie-Hellman assumption in the random oracle model.

### Our Improved AMRIBE Scheme

Tseng et al. [9] proposed an AMRIBE scheme that was proved to be semantically secure against adaptive chosen ciphertext attacks in the random oracle model under the Gap-BDH assumption.

Tseng et al.’s AMRIBE scheme [9] is an extension of Boneh and Franlin’s IBE scheme [3] to multiple recipients scenario. Rapid enhanced-security asymmetric cryptosystems transform (REACT) [26] is an important tool for any asymmetric encryption schemes to achieve IND-CCA secure from IND-CPA secure. We apply the REACT transformation in Tseng et al.’s AMRIBE scheme to further improve the efficiency without compromising security.

- **Setup**: The PKG runs 𝒢(1^*κ*^) to get (*q*, **G**_1_, **G**_2_, *ê*, *P*), chooses $s\overset{\$}{\leftarrow }{\text{Z}}_{q}^{*}$, computes *P*_*pub*_ = *sP* ∈ **G**_1_. Finally, the PKG sets *msk* = *s* and *mpk* = ⟨*q*, **G**_1_, **G**_2_, *ê*, *P*, *P*_*pub*_, *H*_0_, *H*_1_, *H*_2_, *H*_3_, Π⟩.
- **Extract**: Given a user’s identity ID, the PKG computes *Q*_ID_ = *H*_0_(ID) and *d*_ID_ = *sQ*_ID_. Finally, the PKG sends the user’s private key *d*_ID_ to the user via a secure channel.
- **Encrypt**: To encrypt a message *m* ∈ {0, 1}^*^ for *t* receivers with identities $ID={\left\{\text{I}{\text{D}}_{i}\right\}}_{i=1}^{t}$, the sender chooses $r,k\overset{\$}{\leftarrow }{\text{Z}}_{q}^{*}$, computes *U* = *rP*, *T* = *rP*_*pub*_, *Q*_ID_*i*__ = *H*_0_(ID_*i*_) and *v*_*i*_ = *H*_1_(*ê*(*Q*_ID_*i*__, *T*)) for 1 ≤ *i* ≤ *t*, and constructs a polynomial *f*(*x*) with degree *t* as
  $$\begin{array}{c} f\left(x\right)=\prod_{i=1}^{t}\left(x-v_{i}\right)+k\;\mathrm{mod}\;q=c_{0}+c_{1}x+\ldots +c_{t-1}x^{t-1}+x^{t}. \end{array}$$
  The sender then computes *W* = *E*_*H*_2_(*k*)_(*m*) and *λ* = *H*_3_(*m*,*k*,*c*_0_,*c*_1_,…*c*_*t*−1_, *U*, *W*). Finally, the sender sets the ciphertext *C* = 〈*c*_0_, *c*_1_,…,*c*_*t*−1_, *U*, *W*, *λ*〉.
- **Decrypt**: Upon receiving a ciphertext *C* = 〈*c*_0_,*c*_1_,…,*c*_*t*−1_, *U*, *W*, *λ*〉 that is encrypted using identities $ID={\left\{\text{I}{\text{D}}_{i}\right\}}_{i=1}^{t}$, a selected receiver with identity ID_*j*_ ∈ **ID** first computes *v*_*j*_ = *H*_1_(*ê*(*d*_ID_*j*__, *U*)), $k^{\prime }=f(v_{j})=c_{0}+c_{1}v_{j}+\cdots +c_{t-1}v_{j}^{t-1}+v_{j}^{t}\;\text{mod}\;q$ and *m* ^′^ = *D*_*H*_2_(*k*^′^)_(*W*). The receiver then sets *λ* ^′^ = *H*_3_(*m* ^′^, *k* ^′^, *c*_0_, *c*_1_,…*c*_*t*−1_, *U*, *W*), and tests whether *λ* ^′^ = *λ* holds or not. If it does not hold, the recipient rejects the ciphertext. Otherwise, the recipient outputs *m* as the decryption of *C*.

## The AIB-OR Protocol

**Fig 1:**
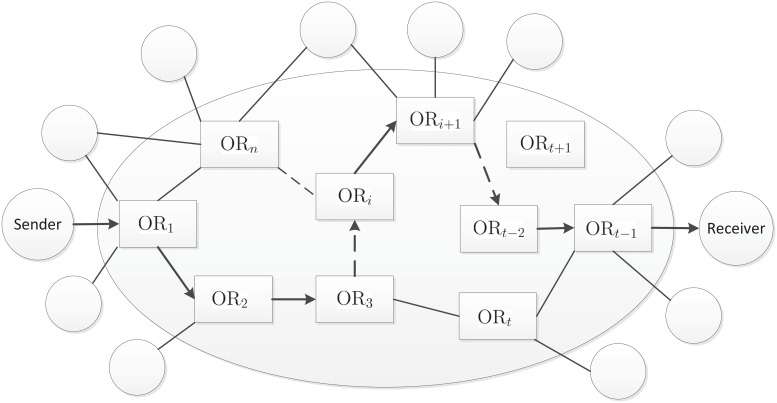
System Model of Onion Routing.

The system model for our proposed AIB-OR protocol is illustrated as Fig. 1, in which the round represents the users and the rectangle represents the onion routers. Assume that there are *t*−1 onion routers between the sender and the receiver along the routing path. We denote the *i*-th router in the path by OR_*i*_ where 1 ≤ *i* ≤ *t*−1. Unlike existing onion routing protocols, we will distinguish users and routers in our AIB-OR.

The proposed AIB-OR protocol involves a trusted PKG whose responsibility is to initialize system parameters and to issue identity-based private keys and blind pseudonym certificates for all participants. The PKG runs the setup algorithm in AMRIBE scheme, sets the master key *msk* = *s*, and publishes the system parameters *mpk* = ⟨*q*, **G**_1_, **G**_2_, *ê*, *P*, *P*_*pub*_, *H*_0_, *H*_1_, *H*_2_, *H*_3_, Π⟩.

### Circuit Construction

When the sender would like to send a message *m* ∈ {0, 1}^ℓ^ to the designated recipient with identity ID_*R*_ anonymously and securely, he performs the following steps:

1. The sender generates his pseudonym PN_*S*_ and gets his pseudonym certificate 〈PN_*S*_, *σ*_*S*_〉 by performing the **PNGen** algorithm and the **BlindCert** algorithm, respectively.
2. The sender runs the **Key Agreement** algorithm of our improved IBOWAKE protocol, gets the session key *K*_*S*, *R*_ = *ê*(*sk*_PN_*S*__, *Q*_ID_*R*__), and encrypts message *m* using a symmetric encryption algorithm (such as Advanced Encryption Standard, AES) with the session key *K*_*S*, *R*_ to get the ciphertext *C*_0_. Note that the seesion key is used to encrypt data and is valid only for the duration of the communication.
3. The sender then constructs a circuit by selecting an ordered subset of onion routers from a generally known set of onion routers. We denote the identities of selected subset of onion routers by ${\left\{\text{I}{\text{D}}_{i}\right\}}_{i=1}^{t-1}$, where ID_*t*−1_ is the identity of onion router closest to the designated receiver.
4. The sender encrypts the inner ciphertext *C*_0_ for identities ${\left\{\text{I}{\text{D}}_{i}\right\}}_{i=1}^{t-1}\cup \text{I}{\text{D}}_{R}$ by applying the **Encrypt** algorithm of our improved AMRIBE scheme to get the outer ciphertext *C*_1_.
5. Finally, the sender transmits the onion packet ONI ≜(*seq*, 〈PN_*S*_, *σ*_*S*_〉, *C*_1_) to the first onion router OR_1_ along the path.

### Decrypt by Onion Router

When an onion router OR_*i*_ receives the onion packet, it processes the onion packet as follows.

1. The onion router OR_*i*_ checks whether the packet has already been received or not by using the field *seq* as the unique identifier for the packet. If there is an entry with the same *seq* field in its local routing table, it simply discards the onion packet. Otherwise it inserts a new record (*seq*, OR_*i*−1_, *ttl*) into the local routing table.
2. The onion router OR_*i*_ decrypts the ciphertext *C*_1_ of the onion packet with its private key *d*_ID_*i*__ by running the **Decrypt** algorithm of our improved AMRIBE scheme. If the decryption fails, the OR_*i*_ just discards the packet without forwarding. Otherwise, the OR_*i*_ forwards the onion packet to all the connecting onion routers and users except the previous onion router (whom it gets the packet from).

### Decrypt by the Recipient

When a user receives the onion packet, he performs the same operations as the onion routers. Note that a user can not decrypt the ciphertext *C*_1_ of the onion packet with his private key unless he is the designated recipient. If he is not the designated recipient, he simply discards the packet. Otherwise, he is the designated recipient, and he can further decrypt the inner ciphertext *C*_0_ with the session key *K*_*S*, *R*_ = *ê*(PN_*S*_, *d*_ID_*R*__) and get the plaintext *m*.

## Security and Efficiency Analysis

**Fig 2:**
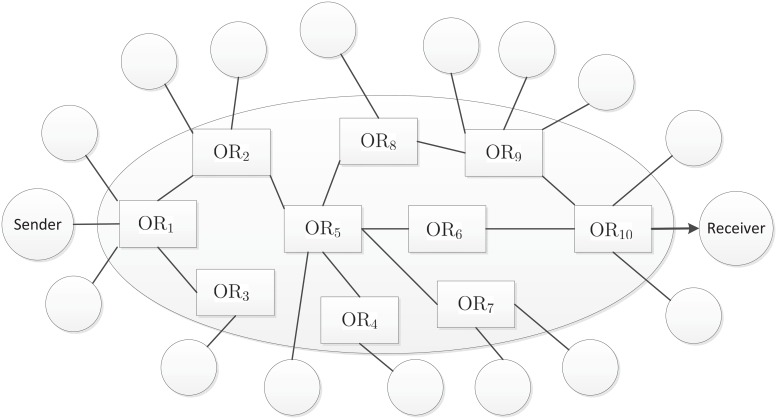
Example of Onion Routing.

In this section, we explain how the proposed AIB-OR protocol meets the sender anonymity, recipient anonymity, route anonymity and failure tolerance, and analyze why the proposed AIB-OR protocol can greatly reduce computational and communications costs. In the following, all analyzes and discussions are based on the onion routing example illustrated in Fig. 2.

- **Sender Anonymity**: The sender constructs circuit using its one-time pseudonym in our AIB-OR protocol, which ensures sender anonymity.
- **Recipient Anonymity**: In existing onion routing protocols, if an adversary compromised the onion router *OR*_10_, then he knows the address or identity of the recipient. In our AIB-OR protocol, the sender encrypts the inner ciphertext for multiple identities ${\left\{\text{I}{\text{D}}_{i}\right\}}_{i=1}^{t-1}\cup \text{I}{\text{D}}_{R}$ by applying AMRIBE scheme, which provides privacy protection for the recipient and the onion routers in the circuit.
- **Route Anonymity**: In the circuit construction given in PB-OR and CL-OR, the sender sends to each onion router a pseudonym and a message symmetrically encrypted with the session key *K*_*i*_. The session key *K*_*i*_ is generated by a non-interactive anonymous key agreement protocol, and the message contains the identity of the next node in the circuit. Thus every onion router in the circuit knows the address or identity of both the previous onion router and the next onion router. In the circuit construction of our AIB-OR protocol, the sender encrypts the inner ciphertext for multiple identities ${\left\{\text{I}{\text{D}}_{i}\right\}}_{i=1}^{t-1}\cup \text{I}{\text{D}}_{R}$ by applying AMRIBE scheme, which ensures none of the onion routers knows who is the next onion router or user since every onion router sends message to all the connecting onion routers and users except the previous onion router.
- **Failure Tolerance**: Assume that sender sends a message to the receiver using the routing path Sender → OR_1_ → OR_2_ → OR_5_ → OR_6_ → OR_10_ → Recipient. If there is something wrong with OR_6_, the message will be discarded after OR_5_ in the existing onion routing protocols. In our AIB-OR protocol, the sender can add both the identity of OR_8_ and OR_9_ to the recipient collection. In this way, both OR_8_ and OR_9_ can decrypt the ciphertext with their own private key, respectively. If OR_6_ failed, the message can still be transferred to the receiver following the path Sender → OR_1_ → OR_2_ → OR_5_ → OR_8_ → OR_9_ → OR_10_ → Recipient. This will bring extra overhead in circuit construction since the sender needs to construct higher order polynomial. Here we assume the sender has sufficient knowledge of routing paths leading to the recipient and the sender make a tradeoff between efficiency and failure tolerance to decide the actual routing path.
- **Message Consistency**: We assume the adversaries have complete control over some part of the network, but not all parts of the network, since this cannot be possible for large network with thousands of network links. It may look like the message is not changing at every hop in the path so this may give path information to an attacker. However, attackers do not know the next hop in the path in our AIB-OR. So there is nearly no possibility for adversaries to get the whole path information by utilizing techniques such as traffic analysis, unless they are watching the entire network. In addition, our AIB-OR, unlike PB-OR and CL-OR, provides message integrity detection by verifying the value of *λ*.
- **Forward Secrecy**: To achieve forward secrecy in PB-OR and CL-OR, onion routers’ keys are required to be changed frequently, this implies a significant computational effort for the PKG. In contrast our AIB-OR, none of the onion routers can decrypt the inner ciphertext and know who is the next onion router or user. To achieve forward secrecy in our proposed AIB-OR, only the sender’s pseudonym or the recipient’s key is required to be changed.
- **Communication Cost**: At first glance, our AIB-OR protocol will bring higher network overhead since each onion router forwards the onion packet to all the connecting onion routers and users except the previous onion router. In fact, out of all only onion routers in the circuit can decrypt and the remaining will discard the onion packet. In Fig. 2, when OR_5_ receives an onion packet from OR_2_, it will send the onion packet to the onion routers OR_4_, OR_6_, OR_7_, OR_8_ and a user, respectively. However, only OR_6_ can decrypt the onion packet and others will discard it since they can not decrypt the onion packet. In addition, the size of the onion packet is shorter than those of PB-OR and CL-OR.
- **Storage Cost**: Both onion routers and users are given an identity-based private key from the trusted PKG, which can be used to decrypt the ciphertext encrypted by AMRIBE scheme as well as symmetric encryption algorithm with the session key *K*_*S*, *R*_, thus the overhead of key management is greatly reduced. In addition, for an onion router or a user who receives the same packet for the second time, they can check the field *seq* in the packet against entries in the local routing table. If there is a matching record in the routing table, the onion router or user will discard the packet. A time to live field (*ttl*) is set in the local routing table, so that the entry can be removed from the local routing table when the *ttl* reaches zero.
- **Computation Cost**: In the PB-OR and CL-OR, a sender is required to perform symmetric encryption operation *t* times if there are *t* onion routers in the path. In our AIB-OR, a sender is only required to perform symmetric encryption operation 2 times irrespective of number of onion routers in the path. Our AIB-OR, like PB-OR and CL-OR, each intermediate onion router is required to perform one bilinear pairing and one symmetric decryption operations. Note that, message is actually transmitted from the last onion router to the designated recipient in the clear text both in the PB-OR and CL-OR. Thus, the recipient does not need to perform any operations in PB-OR and CL-OR. Obviously, this would lead to the disclosure of messages and exposure of recipient identity. Our AIB-OR, unlike existing onion routing protocols, the user and the onion router is different, and the recipient is required to perform two bilinear pairing and two symmetric decryption operations.

We compare the security properties, computational and communications costs on circuit construction in PB-OR, CL-OR and our AIB-OR. The comparison is summarized in Table 2. We denote by *t*, |*m*|, |ID|, |**G**_1_| and $\left|{\text{Z}}_{q}^{*}\right|$ the number of onion routers in the circuit, the bit-length of a plaintext, an identity, an element in group **G**_1_, and an element in group ${\text{Z}}_{q}^{*}$, respectively. We denote by *e*_1_, *e*_2_, *p*, *E*, *D* the computation cost of an exponentiation in **G**_1_, an exponentiation in **G**_2_, a bilinear pairing in (**G**_1_, **G**_2_), a encryption operation and a decryption operation in Π, respectively. Other operations are omitted in the following analysis since their computation cost is trivial.

**Table 2:** Comparison among our AIB-OR, PB-OR and CL-OR.

	PB-OR [10]	CL-OR [12]	AIB-OR
Sender Cost	*tp*+*tE*+*te*_1_+*te*_2_	*tE*+5*te*_1_	(*t*+1)*p*+2*E*+2*e*_1_
OR Cost	1*p*+1*D*	3*e*_1_+1*D*	1*p*+1*D*
Onion Packet Size	|*m*|+(*t*−1)|ID|+*t*|**G**_1_|	$\left|m|+(t-1)|\text{ID}|+t|{\text{Z}}_{q}^{*}\right|$	$\left|m|+(t+1)|{\text{Z}}_{q}^{*}|+|{\text{G}}_{1}\right|$
Recipient Anonymity	No	No	Yes
Sender Anonymity	Yes	Yes	Yes
OR Anonymity	Partial	Partial	Complete
Fault Tolerance	No	No	Yes
Forward Secrecy	Yes	Yes	Yes
Message Integrity	No	No	Yes
MIMA Resistance	No	No	Yes

## Performance Test

We conducted several experiments to compare AIB-OR with PB-OR in terms of computation cost and bandwidth overhead. The experiments were run on a machine with 2GB of RAM, and hosted on 2.00GHz. We implement our algorithms based on Charm-Crypto Framework (version 0.42) [27] and Pairing-Based Crypto (PBC) library [28].

In our experiment, we use symmetric bilinear groups over supersingular elliptic curves of type A [28], where an element in **G**_1_ can be represented by 512 bits. We choose AES-256 as the symmetric encryption algorithm, and the number of onion routers is chosen to be from 5 to 20, and the packet size is chosen to be 512 bytes.

**Fig 3:**
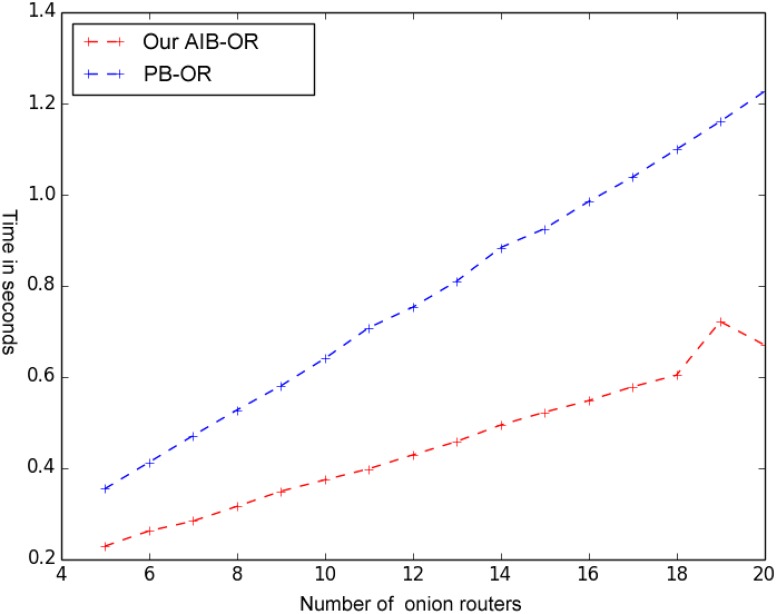
Comparison of Circuit Construction Cost.

The time cost of circuit construction is measured on encryption time run by the source node, decryption time run by all the intermediate nodes in the circuit and decryption time run by the destination node. Fig. 3 shows the comparison of computation time required by the sender in PB-OR [10] and our AIB-OR. As shown in the figure, the computation time required by the sender in our AIB-OR is shorter than in PB-OR for the same number of onion routers. Moreover, the growth rate of computation time required by the sender in our AIB-OR is slower than in PB-OR.

**Fig 4:**
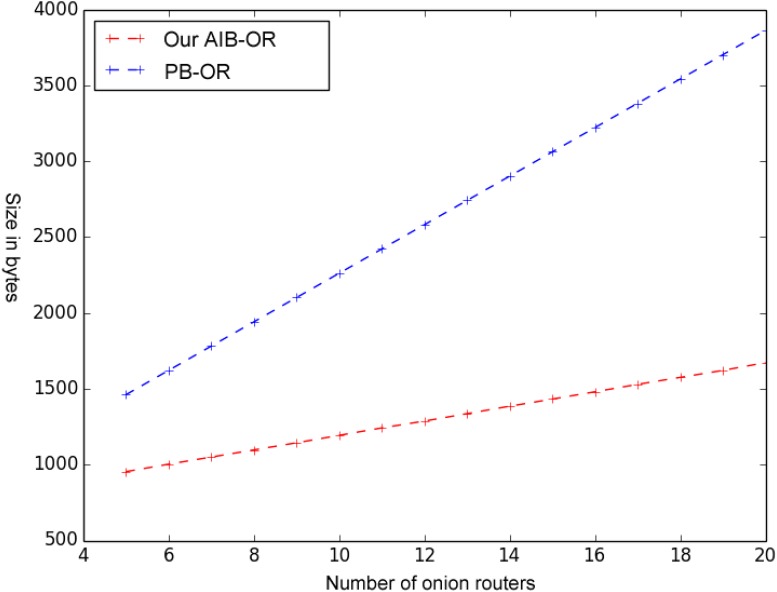
Comparison of Onion Packet Size.

For PB-OR, the size of onion packets becomes larger with the increase in the number of onion routers. For the intermediate routers, the closer to the destination node, the higher bandwidth overhead will be. There is no such problem in our AIB-OR. Fig. 4 shows the comparison of onion packet size in PB-OR [10] and in our AIB-OR. As shown in the figure, the size of onion packet in our AIB-OR is shorter than in PB-OR. Moreover, the growth rate of onion packet size in our AIB-OR is slower than in PB-OR.

## Conclusion

In this paper, we propose a new approach for circuit construction in onion routing anonymity networks by using our improved anonymous multi-receiver identity-based encryption scheme and our improved anonymous identity-based one-way key agreement protocol. Compared to existing approach for circuit construction in onion routing anonymity networks, our approach provides high efficiency, scalability, strong anonymity and fault tolerance. Performance experiment shows that our proposed approach uses significantly less computation and communication than that of paring-based onion routing.
